# Motor Timing Deficits in Sequential Movements in Parkinson Disease Are Related to Action Planning: A Motor Imagery Study

**DOI:** 10.1371/journal.pone.0075454

**Published:** 2013-09-23

**Authors:** Laura Avanzino, Elisa Pelosin, Davide Martino, Giovanni Abbruzzese

**Affiliations:** 1 Department of Experimental Medicine, Section of Human Physiology and Centro Polifunzionale di Scienze Motorie, University of Genoa, Genoa, Italy; 2 Department of Neuroscience, Rehabilitation, Ophthalmology, Genetics, Maternal and Child Health, University of Genoa, Genoa, Italy; 3 Queen Elizabeth Hospital, South London NHS Trust, London, United Kingdom; 4 King’s College Hospital, London, United Kingdom; 5 Centre for Neuroscience and Trauma, Queen Mary University of London, London, United Kingdom; University Medical Center Groningen UMCG, Netherlands

## Abstract

Timing of sequential movements is altered in Parkinson disease (PD). Whether timing deficits in internally generated sequential movements in PD depends also on difficulties in motor planning, rather than merely on a defective ability to materially perform the planned movement is still undefined. To unveil this issue, we adopted a modified version of an established test for motor timing, i.e. the synchronization–continuation paradigm, by introducing a motor imagery task. Motor imagery is thought to involve mainly processes of movement preparation, with reduced involvement of end-stage movement execution-related processes. Fourteen patients with PD and twelve matched healthy volunteers were asked to tap in synchrony with a metronome cue (SYNC) and then, when the tone stopped, to keep tapping, trying to maintain the same rhythm (CONT-EXE) or to imagine tapping at the same rhythm, rather than actually performing it (CONT-MI). We tested both a sub-second and a supra-second inter-stimulus interval between the cues. Performance was recorded using a sensor-engineered glove and analyzed measuring the temporal error and the interval reproduction accuracy index. PD patients were less accurate than healthy subjects in the supra-second time reproduction task when performing both continuation tasks (CONT-MI and CONT-EXE), whereas no difference was detected in the synchronization task and on all tasks involving a sub-second interval. Our findings suggest that PD patients exhibit a selective deficit in motor timing for sequential movements that are separated by a supra-second interval and that this deficit may be explained by a defect of motor planning. Further, we propose that difficulties in motor planning are of a sufficient degree of severity in PD to affect also the motor performance in the supra-second time reproduction task.

## Introduction

The subjective representation of the passage of time is critical for a variety of motor activities. When planning a complex motor action, the central nervous system should execute an accurate integration of temporal as well as spatial information. The neural network supporting motor timing comprises the lateral cerebellum, basal ganglia, and sensorimotor cortical areas [1-4].

Particularly, the basal ganglia and their associated subcortical dopaminergic system play a crucial role acting as a hypothetical “internal clock” that beats the rhythm when the movement is internally generated [5,6]. The role of the basal ganglia in timing is particularly relevant to individuals with idiopathic Parkinson’s disease (PD), who exhibit temporal processing deficits [5,7] that may contribute to the breakdown in the spatiotemporal patterning of movements. Bradykinesia (slowness of movement initiation and execution), a cardinal symptom of PD, is particularly evident for internally generated sequential movements, and can benefit from the introduction of external rhythmic cues [8-10].

Whether timing deficits in internally generated sequential movements in PD depends also on difficulties in motor planning rather than merely on a defective ability to materially perform the planned movement is still undefined. Motor imagery corresponds to the mental rehearsal of a movement without overtly performing the respective action [11-13], and is thought to involve mainly processes of movement preparation, with reduced involvement of end-stage movement execution-related processes [14,15].

Abnormal performance on motor imagery tasks has been demonstrated in patients with PD using different approaches, including behavioural, electrophysiological (transcranial magnetic stimulation and movement-related potentials) and functional imaging studies [16-19]. These studies have also highlighted changes in functional activation of circuits interconnecting frontal cortical areas and basal ganglia in relation to motor imagery tasks in PD patients, further supporting general abnormalities of motor planning in this condition [16,18,20-22].

Despite this body of evidence, the contribution of motor planning abnormalities to the performance of internally generated sequential movements has never been directly explored through the analysis of the temporal features of movement in PD. To shed more light on this aspect, we adopted a modified version of an established test for motor timing, i.e. the synchronization-continuation paradigm, by introducing a motor imagery task. The synchronization-continuation paradigm involves: i) a synchronization phase, in which subjects are asked to tap in synchrony with a train of tones separated by a constant inter-stimulus interval (ISI), and ii) a continuation phase, in which subjects are requested to continue tapping at the previous rate in the absence of the auditory cue. The addition of a motor imagery task to the classical synchronization-continuation paradigm aims at disentangling, within the entire process of sequential finger movement production (the “classical” continuation task), the phase of motor planning from that of movement execution. In the present study, during the continuation phase patients with PD and healthy volunteers were asked, on the basis of the information received and stored during the synchronization phase, to either materially perform the movement (execution task) or imagine performing it (imagery task). Further, since performance on the synchronization-continuation test is largely dependent on the duration of the inter-stimulus interval (ISI), we tested both a sub-second (metronome rate: 1.5 Hz, ISI: 666 ms) and a supra-second (metronome rate: 0.5 Hz, ISI: 2000 ms) inter-stimulus interval between the cues.

## Materials and Methods

### Ethical Statement

All participants gave their written informed consent prior to their inclusion in this study. The experimental protocol was approved by the ethics committee of the University of Genoa (Protocol nr. 31/12) and was carried out in agreement with legal requirements and international norms (Declaration of Helsinki, 1964).

### Subjects

Fourteen patients with PD (8 males; mean age 68.78 ± 8.71 SD years) were recruited from the outpatient Movement Disorders Clinic of the IRCCS, San Martino Hospital, University of Genoa. Demographic and clinical information for patients with Parkinson’s disease are reported in Table 1. Twelve aged-matched healthy subjects (HS, 7 males; mean age 64.15 ± 10.88 years) with normal neurological examination and no history of neurological disorders were recruited as control subjects from hospital staff or patients’ spouses or friends. In the PD group, disease severity was determined using the Motor part (III) of the Unified Parkinson’s Disease Rating Scale (UPDRS). Inclusion criteria for the patients’ group were: a diagnosis of PD according to the UK Parkinson’s Disease Society Brain Bank criteria; Hoehn & Yahr stages 1-3; stable dopaminergic medication regimen. Exclusion criteria were a history of any neurological disease other than PD; ongoing functional brain surgery treatment; Mini-Mental State Examination corrected score < 24; visual or hearing impairment; severe orthopedic problems of the upper limb. All PD subjects were taking levodopa alone or combined with a dopamine agonist and were tested in their ‘ON’ dopaminergic state (PD subjects took their normal daily medication dosage one hour before the experimental session). All participants were right-handed except for two in the control group. Right hand dominance has been evaluated by the Edinburgh Handedness Inventory [23]. In all subjects with PD the more affected arm was tested, while in healthy participants the test was performed on the dominant hand.

**Table 1:** Demographic and clinical information for patients with Parkinson’s disease.

**#**	**AGE**		**GENDER**	**DISEASE (years**)	AFFECTED SIDE*	**HY**	**UPDRS (III**)
1		75	M	13	right	2,5	30
2		48	F	3	right	1	11
3		63	F	2	left	2	21
4		74	M	3	right	2	22
5		68	M	1	right	2	24
6		75	F	13	right	2	13
7		73	M	4	right	2	31
8		65	F	8	left	2	32
9		78	M	10	left	2,5	37
10		55	F	2	left	1	5
11		76	F	3	left	2	16
12		67	M	9	right	2	14
13		70	M	4	right	2	24
14		76	M	1	right	2	20

HY: Hoehn and Yahr scale; UPDRS: Unified Parkinson’s disease Rating Scale

* the more affected side is indicated in the table

### Motor task

Subjects were seated in a comfortable chair in a quiet and darkened room. They wore a sensor-engineered glove (eTT, Genova, Italy) on their dominant hand (HS) or their most affected side (PD). Data were acquired at 1 KHz. An eyes closed paradigm was chosen to avoid possible confounding effects due to the integration of acoustic and visual information. The experimental motor task (sequential opposition of thumb to index, medium, ring and little fingers) was shown to the participants through a video-clip.

**Figure 1:**
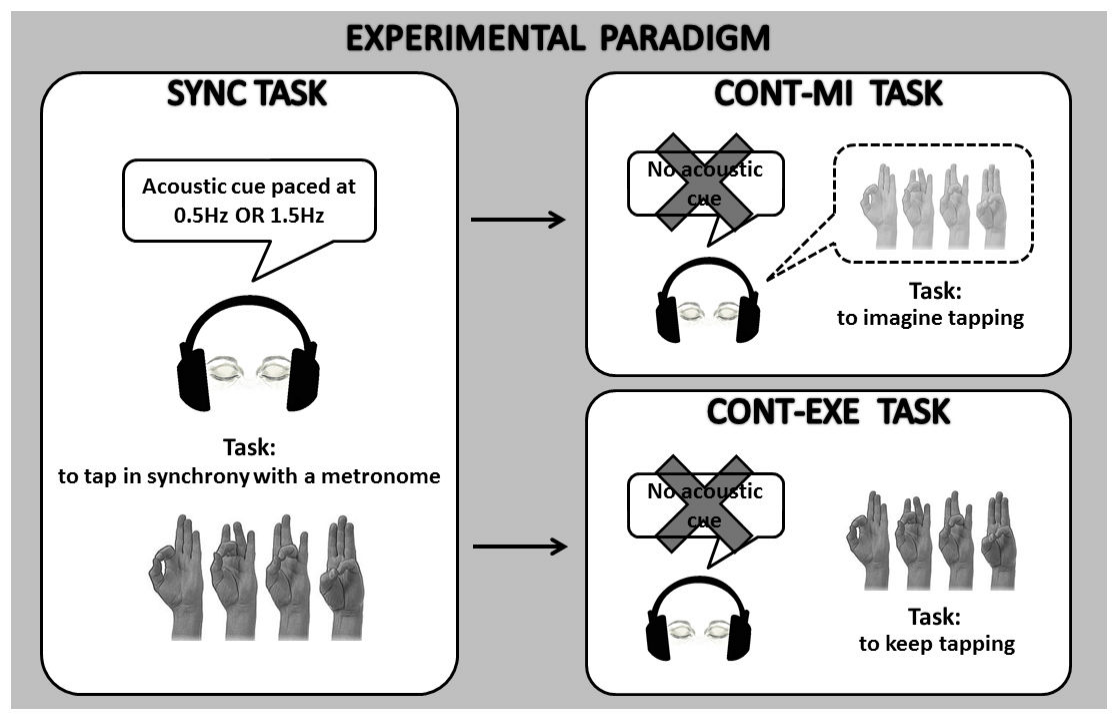
Experimental paradigm. In our modified version of a synchronization-continuation task, subjects were requested to tap in synchrony with a metronome cue (SYNC) and then, when the tone stopped, to tap the fingers in a sequential order, trying to maintain the same rhythm as accurately as possible (CONT-EXE) or to imagine finger tapping at the same rhythm, rather than actually performing it (CONT-MI). The order of the tasks was random.

Subjects completed two tasks: 1) the execution task, in which they were requested to tap in synchrony with a metronome cue (SYNC) and subsequently, when the tone stopped, to tap the fingers in a sequential order, trying to maintain the same rhythm as accurately as possible (CONT-EXE); 2) the motor imagery task, in which, after the SYNC phase, participants were requested, during the continuation phase, to imagine finger tapping at the same rhythm, rather than actually performing it (CONT-MI). In order to approximately calculate the duration of the “imagined” time interval (see data analysis section), participants were instructed to oppose the thumb to the first finger of the sequence (index) and then imagine the rest of the sequence, opposing again the thumb to the index finger at the start of the following imagined sequence. For the motor imagery task, subjects were asked to imagine themselves executing the movement (‘internal imagery’) rather than watching themselves performing it (‘external imagery’). Each phase (SYNC and CONT) lasted 45 seconds. Two blocks for each task (execution and motor imagery) were performed in random order with a different metronome pace (1.5 Hz, i.e. time interval between two successive metronome cues: 666 ms; 0.5 Hz, i.e., time interval: 2000 ms). The metronome pace values were chosen in order to have one sub-second time interval (666 ms) and one supra-second time interval (2000 ms) between two successive auditory stimuli to be reproduced in the CONT-EXE and CONT-MI tasks. Figure 1 summarizes the experimental protocol.

### Data analysis

Data were processed with a customized software (GAS, eTT, Genoa, Italy) that extracts the duration of the time interval between two successive finger contacts (in ms). In the CONT-EXE task, this interval corresponded to the time interval reproduced by the subjects. In the CONT-MI task, since subjects were asked to oppose the thumb to the first finger of the sequence (index), imagine the rest of the sequence (thumb to medium, thumb to ring and thumb to little fingers) and oppose again the thumb to the index finger at the start of the following imagined sequence, the time interval calculated by GAS corresponded to an entire cycle sequence. Therefore, in order to approximately calculate the time interval between two subsequent imagined finger contacts, the value obtained was divided by the number of opposition movements of an entire sequence (four tapping movements).

Performance on the execution and motor imagery tasks was analyzed by measuring the temporal error and the interval reproduction accuracy index. The temporal error corresponds to the duration of the time interval reproduced by the subject minus the duration of the time interval set by the metronome, and provides a direct measure of the magnitude of the error in reproducing the corresponding time interval (in ms). The interval reproduction accuracy index is the ratio between the time interval reproduced by the subject and the time interval set by the metronome, and allows a comparison of performance at each time interval, independent of duration; this index provides also the directionality of the tapping performance, being >1 if the participant is behind the beat and <1 if the participant is ahead of the beat.

### Statistical Analysis

Since data were normally distributed (according to the Kolmogorov Smirnov statistical test), we used parametric tests. Temporal error and interval reproduction accuracy index were analyzed by means of a repeated measures analysis of variance (RM-ANOVA) with GROUP (patients with PD, healthy subjects) as between-subjects factor and TASK (SYNC, CONT-EXE and CONT-MI) and TIME INTERVAL (666 and 2000 ms) as within-subjects factors. *Post hoc* analyses of significant interactions were performed using *t*-tests applying the Bonferroni correction for multiple comparisons where necessary. P values lower than 0.05 were considered as threshold for statistical significance. Finally, Spearman’s correlation coefficients were calculated to assess any correlation between ability in time reproduction parameters and disease severity. Statistical analysis was performed with SPSS 13.0.

## Results

### Temporal Error

**Figure 2:**
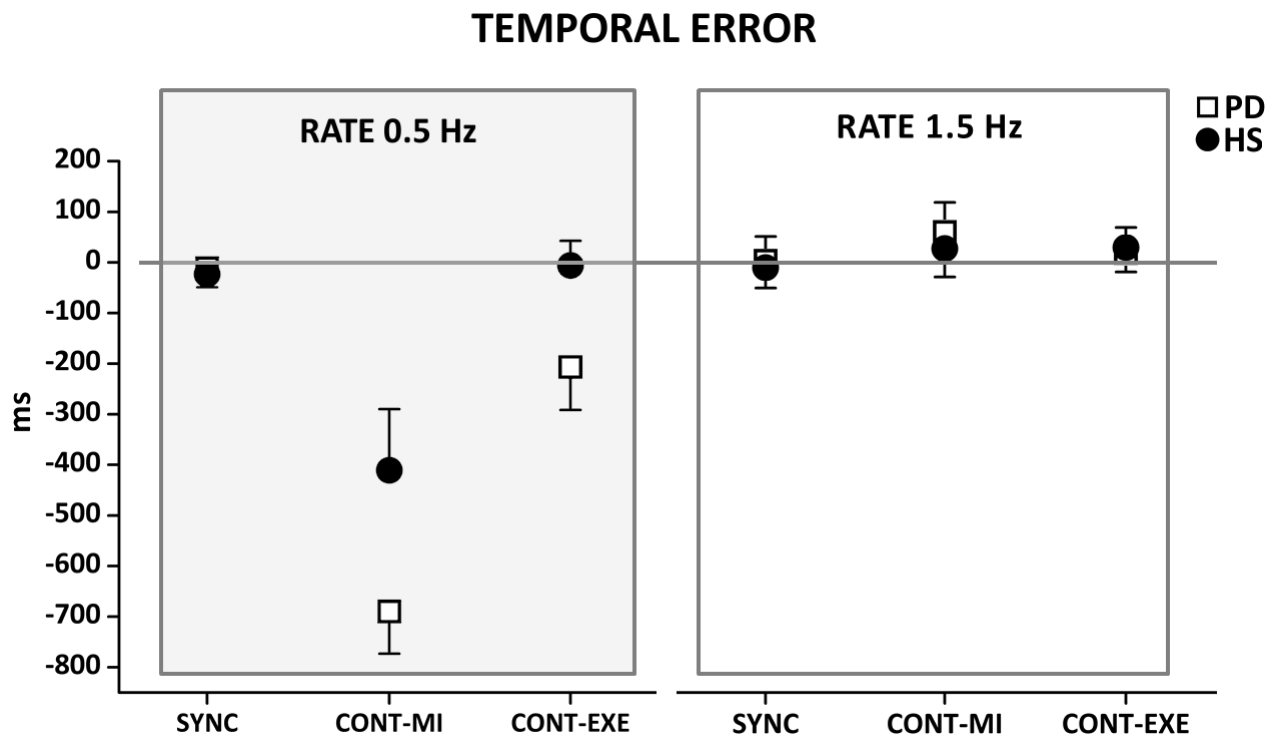
Temporal error expressed in ms. Data of both patients with Parkinson’s disease (PD) and healthy control subjects (HS) are shown. The results of the synchronization (SYNC), execution (CONT-EXE) and motor imagery (CONT-MI) tasks with a supra-second (0.5 Hz) and sub-second (1.5 Hz) time interval are shown. On the x-axis, we show the type of task. On the y-axis, we show the duration of the temporal error. Asterisk indicates differences between PD and HS when interaction of TIME INTERVAL*GROUP* TASK was statistically significant (*p<0.05). Mean data + standard error mean (SEM) are shown.

Analysis of variance showed that the size of the temporal error in our paradigm was influenced by different within- and between-subjects factors (Figure 2). First of all, a significant effect of TIME INTERVAL was found (F[1,24]= 44.86; p< 0.001); the temporal error was larger in both PD patients and HS in the supra-second time interval (2000 ms) task compared to the sub-second one (666 ms). Second, a significant effect of TASK was detected in both groups (F[2,48]= 24.93; p< 0.001); there was a larger temporal error in the CONT-MI compared to the SYNC (p<0.001) and the CONT-EXE (p<0.001) tasks, with no difference between the SYNC and the CONT-EXE tasks (p=0.29). We observed also a significant effect of the TIME INTERVAL*TASK interaction (F[2,48]= 41.00; p< 0.001); post hoc analyses showed that the temporal error was similar between CONT-MI and CONT-EXE in the sub-second time reproduction task (p=0.31), whereas temporal error was larger in CONT-MI with respect to CONT-EXE in the supra-second time reproduction task (p<0.001). Finally, a significant effect of the TIME INTERVAL*GROUP*TASK interaction was found (F[2,48]= 2.66; p= 0.045); post hoc analyses revealed that the temporal error was significantly larger in PD patients than in HS in the supra-second time interval task (2000 ms, 0.5 Hz) in both the CONT-MI (p= 0.04) and CONT-EXE (p=0.045), but not in the SYNC tasks (p= 0.79). Also, although in the supra-second time interval task the temporal error was similar between CONT-EXE and SYNC in HS (p=0.82), this parameter was still significantly larger in CONT-EXE with respect to SYNC (p= 0.012) in PD patients. No difference between PD patients and HS was detected in the sub-second time interval task.

### Interval reproduction accuracy index

**Figure 3:**
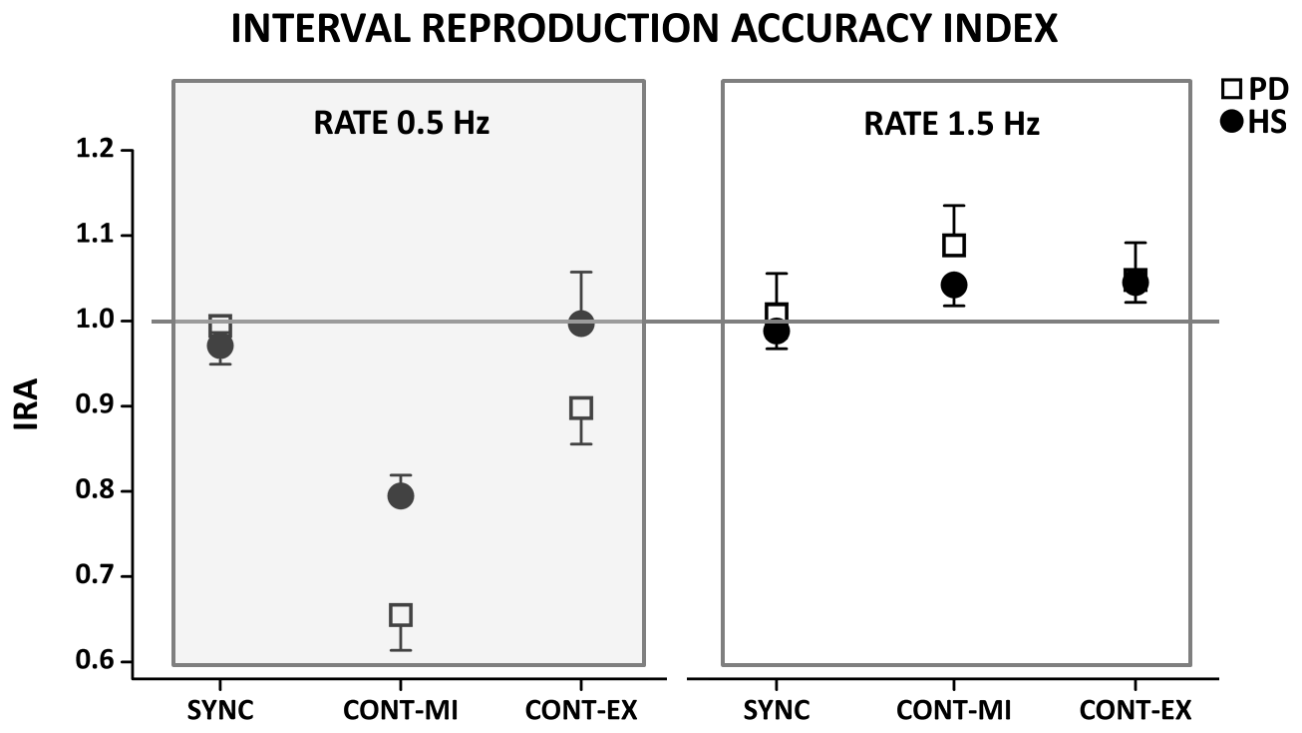
Interval reproduction accuracy index (IRA). Data of both patients with Parkinson’s disease (PD) and healthy control subjects (HS) are shown. The results of the synchronization (SYNC), execution (CONT-EXE) and motor imagery (CONT-MI) tasks with a supra-second (0.5 Hz) and sub-second (1.5 Hz) are shown. On the x-axis, we show the type of task. On the y-axis, we show the IRA expressed as a ratio between the time interval reproduced by the subject and the time interval set by the metronome. Asterisk indicates differences between PD and HS when interaction of TIME INTERVAL*GROUP* TASK was statistically significant (*p<0.05). Mean data + standard error mean (SEM) are shown.

Similar to the temporal error, the interval reproduction accuracy index was influenced by different factors (Figure 3). A significant effect of TIME INTERVAL was observed (F[1,24]= 39.38; p< 0.001), whereby both PD patients and HS were more accurate when requested to reproduce a sub-second time interval (666 ms) than when requested to reproduce a supra-second time interval (2000 ms). Second, TASK exerted a significant effect (F[2,48]= 11.82; p< 0.001), whereby in both PD and HS groups the performance on the CONT-MI task was less accurate than that recorded on the SYNC (p=0.001) and on the CONT-EXE (p=0.001) tasks, without any difference between the latter two tasks (p=0.78). We also observed a significant effect of the TIME INTERVAL*TASK interaction term (F[2,48]= 43.82; p< 0.001), and post hoc analyses revealed that accuracy in the supra-second time reproduction task was reduced during CONT-MI with respect to CONT-EXE (p<0.001), whereas in the sub-second time reproduction task there was no difference between CONT-MI and CONT-EXE (p= 0.37). Finally, a significant effect of the TIME INTERVAL*GROUP*TASK interaction was found (F[2,48]= 4.39; p= 0.018); post hoc analyses revealed that patients with PD were less accurate than HS when requested to reproduce a supra-second time interval (2000 ms, 0.5 Hz) in both the CONT-MI (p= 0.026) and CONT-EXE (p=0.045) tasks but not on the SYNC task (p= 0.47). Also, although in the supra-second time interval task the interval reproduction accuracy was similar between CONT-EXE and SYNC in HS (p=0.62), this parameter was still significantly larger in CONT-EXE with respect to SYNC (p=0.011) in PD patients. No difference between PD patients and HS was found in the accuracy on the sub-second time interval reproduction task.

### Correlation with clinical features

Given that RM-ANOVA showed that PD patients exhibited a larger temporal error and a smaller interval reproduction accuracy index than HS in the supra-second time interval reproduction task, both when only imagined (CONT-MI) and when really executed (CONT-EXE), we analysed whether these time reproduction parameters correlated with disease duration and severity. Our correlation analyses showed that both the supra-second interval temporal error (CONT-MI: R= -0.10, p= 0.71; CONT-EXE: R= -0.35, p= 0.21) and the interval reproduction accuracy index (CONT-MI: R= -0.10, p= 0.71; CONT-EXE: R= -0.39, p= 0.17) did not correlate with disease severity, suggesting that the performance on these tasks is independent from the severity of motor symptoms. Likewise, both the supra-second interval temporal error (CONT-MI: R= -0.018, p= 0.95; CONT-EXE: R=0.11, p= 0.69) and the interval reproduction accuracy index (CONT-MI: R=-0.018, p= 0.95; CONT-EXE: R=0.10, p= 0.72) did not correlate with disease duration.

## Discussion

In the present study, we adopted a modified synchronization-continuation task in order to investigate whether motor timing deficits in sequential movements in Parkinson’s disease depend on difficulties in motor planning or on the ability to implement the motor plan when subjects materially execute sequential finger opposition movements. Our task was an explicit motor timing task, where the ‘task goal’ was to provide an accurate estimate of elapsed time. During the metronome phase, subjects stored temporal information related to an auditory stimulus presented at regular inter-stimulus intervals (ISI). During the imagery and execution phase, subjects respectively used the temporal information acquired to imagine or actually reproduce a motor representation of the timed ISI, in the absence of the sensory pacing stimulus.

The main finding of our study is that PD patients were less accurate than healthy individuals in the supra-second time reproduction task when performing the continuation tasks (CONT-MI and CONT-EXE), whereas no difference was detected in the synchronization task (SYNC) and on all tasks involving a sub-second interval. Also, in the supra-second time reproduction task both PD patients and healthy individuals exhibited a marked drop in reproduction accuracy on the CONT-MI task compared to the SYNC and CONT-EXE tasks, but only PD patients still exhibited an abnormal performance on the CONT-EXE task with respect to SYNC. Given that PD patients have the ability to execute the sequential task with the same accuracy as healthy subjects when this is synchronized with a metronome, these results may be explained by a defect of motor planning of internally generated sequential movements separated by a supra-second time interval, which is of a sufficient degree of severity to affect both the CONT-MI and the CONT-EXE tasks.

Regardless of the presence of PD, the ability to mentally reproduce a certain time interval between sequential finger movements was influenced by the duration of the time interval. Indeed, all subjects were more accurate in internally reproducing the sub-second time interval than the supra-second one. In particular, no difference in accuracy was found between the imagination (CONT-MI) and execution (CONT-EXE) tasks when the time interval was sub-second, while, when the time interval was supra-second, there was a decrease in temporal accuracy and a larger temporal error in the CONT-MI task with respect to the CONT-EXE. In other words, if a perfect isochrony between imagined and really executed movements was found when the ISI between two successive finger opposition movements was sub-second, this was not the case when the interval was supra-second.

Motor imagery has been associated with planning stages of motor production, and, in particular, with internal models that predict the sensory consequences of motor commands and specify the motor commands required to achieve a given outcome. These internal models are modified according to practice and experience, and are requisite for motor learning and for the generation of skilled actions [24-26]. We interpret our results on the basis of the assumption that the ability to mentally simulate a certain movement is strictly linked with the matching of the movement with the individual’s personal *motor repertoire*. As an example, Fourkas and coworkers [27] demonstrated that expert tennis players utilized their imagery, in particular the kinesthetic aspect, more effectively than novices but only for the activity in which they had expertise. Over the entire life span, with respect to finger tapping movements, spontaneous movement tempo (the inter-stimulus interval between two successive unpaced tapping movements) shifts from approximately 300 ms for young children (ages 4–7) to nearly 700 ms for adults aged 75 and over [28]. This evidence suggests that the accuracy in mentally simulating sequential tapping movements may increase as the tapping rate gets closer to the individual’s spontaneous movement tempo. Here, participants were indeed more accurate when requested to imagine tapping movements separated by 666 ms ISI with respect to 2000 ms, with 666 ms ISI really close to the spontaneous movement tempo for non-paced tapping described by McAuley in an adult population [28].

Moreover, by manipulating the time interval between two consecutive finger movements from a sub-second to a supra-second one, we considerably increased the cognitive load of the task, which became more cognitively controlled. At difference, temporal processing of a 500 ms interval or shorter is supposedly of a highly perceptual nature, fast, parallel and not accessible to cognitive control [29]. We acknowledge the possibility that the difference in the attention and working memory demand between the two tasks (sub and supra-second) might have influenced motor imagery ability detected in both our PD patients and healthy subjects in the supra-second time reproduction task [30,31].

Finally, recent data present in the literature suggest that motor imagery ability may decline with ageing. Specifically, mental simulation of more complex or unusual motor tasks was strongly influenced by age [32-34]. In the present study we recruited an elderly population and therefore the observed effect of a decline in motor imagery ability when subjects were asked to mentally reproduce a supra-second time interval between sequential finger movements may be also explained by the higher complexity of the task.

Regardless of the mechanisms leading to abnormal motor imagery ability in our PD patients and elderly subjects, to our knowledge this is the first demonstration of how the temporal features of the “imagined movement” may influence motor imagery accuracy. This result fit with evidence in the literature showing that motor imagery ability increases as the spatial characteristics of the imagined movements become more similar to the normal biomechanical constraints of real movements [35-37]. All these findings may be explained within the theoretical framework of embodiment, viewing motor imagery as a profound body-based simulation process that uses the motor system as a substrate [38].

Existing data on time processing in PD patients present inconsistencies depending on the type of time processing task implemented (time estimation, time production, time reproduction), the time intervals used [39] and on the clinical characteristics of the patients [5,40,41]. However, our observation that sub-second time processing is not impaired in PD is in agreement with previous studies suggesting that structures other than the basal ganglia (e.g. the cerebellum) may provide the representation of the precise timing of events in the millisecond range [42-44]. Differently, paced finger tapping tasks at supra-second intervals were found to be performed at a lower degree of accuracy in PD patients, and this was associated with reduced activation within sensorimotor cortex and supplementary motor area on functional imaging [45]. The selective difference observed with supra-second time intervals in our work, is suggestive of a specific defective involvement of networks inter-connecting the striatum, the dorsolateral prefrontal cortex and the supplementary motor area [2,46-48]. This hypothesis is further supported by the observation that a defective ability in time processing of supra-second time intervals in PD patients was detected in the imagery task, as well as in the execution task. Among the activated brain areas during motor imagery, the supplementary motor area was reported to be the most active area and plays an important role in motor imagery tasks as well as in high-level motor control [49-54]. In PD, PET studies confirmed relative reduction in the activation of the supplementary motor area, the dorsolateral prefrontal cortex and basal ganglia during motor imagery tasks [17,18]. The study of early-stage pre-movement activity (arising mainly from mesial frontal regions) using cortical potentials showed decreased amplitude in PD patients when they imagined movement and correlated with disease severity, whereas movement execution-related components (arising mainly from the primary motor cortex) were relatively unaffected [16].

Based on the above considerations on the functional anatomical basis of the observed changes, we can assume that PD patients have difficulties in planning the temporal characteristics of sequential finger movements when the inter-stimulus interval between two consecutive movements is in the supra-second range, and that difficulties in motor planning are reflected in abnormal motor performance. We can also hypothesize, on the basis of the evidence present in the literature regarding the neural substrates of motor imagery, that this deficit may depend upon an abnormal connection between basal ganglia and interconnected cortical areas, including the supplementary motor area. Further, we propose that difficulties in motor planning are of a sufficient degree of severity in PD to affect also the real execution of the supra-second time reproduction task. Indeed, PD patients exhibited an abnormal performance not only in CONT-MI, but also on the CONT-EXE task with respect to SYNC, whereas HS did not.

One limitation of the present study is that PD patients were tested only in their ‘ON’ state of medication. However, it has been recently demonstrated that PD patients treated with dopamine replacement therapy (whether tested in the ‘ON’ or ‘OFF’ conditions) performed differently in the synchronization-continuation test than early untreated PD patients and healthy controls, with no particular effect of the dopaminergic state [55]. In addition, the lack of correlation between timing accuracy and clinical variables directly related to the illness, such as severity score, may suggest that this abnormal feature is not a direct expression of the severity of motor symptoms.

Our experimental paradigm was aimed at disentangling, within the entire process of sequential finger movement production (the “classical” continuation task), the phase of motor planning from that of movement execution. Thus, we can only assume that PD patients have deficit in planning the temporal features of sequential finger movements when the interval between two successive movements is supra-second, but we cannot discern whether this represents a distorted mental representation of time or, rather, defective spatio-temporal integration during motor planning. The data present in the literature so far support both interpretations, showing that PD patients are impaired in pure time processing tasks [56,57], but also in sensorimotor integration processes [58,59]. However an ad hoc study designed to correlate perceptual timing with motor planning ability in patients with PD might be useful to shed more light on the function of basal ganglia and interconnected cortical areas on this issue. Also, studies devoted to investigate the neural networks (likely comprehending the basal ganglia and the cortical interconnected areas) involved in this task should clarify the physiological basis of timing deficits in internally generated sequential movements in PD that are strictly linked to core symptoms of the disease (e.g. bradykinesia).

Finally, it would be interesting to evaluate the ability in planning the temporal features of sequential movements by means of our motor imagery paradigm in patients affected by neurological diseases other than PD. Indeed, motor imagery is a complex cognitive process that consistently recruits a large fronto-parietal network in addition to subcortical (basal ganglia) and cerebellar regions [60]. Evidence supporting this assumption arises from behavioral and imaging studies carried out in stroke patients with cerebellar or cortical damage. Structural and functional connectivity in parietofrontal pathways, both ipsi- and contralateral to the lesion, determines neural modulation associated with grasping imagery after stroke [61]. After parietal [62] and cerebellar [63,64] damage, patients showed reduced ability in motor imagery processes, with slowing in motor imagery, strongly supporting the notion that these neural structures are important for the ability to generate mental representations of movement. This piece of knowledge would have a strong impact in the field of neurorehabilitation where motor imagery is currently getting a foothold. Indeed, in the present work, we highlighted, for the first time, how the temporal features of the imagined movement can influence motor imagery ability in PD patients. We think that this aspect has to be carefully taken into consideration when designing rehabilitative protocols based on motor imagery.
